# How Does the Central Nervous System for Posture and Locomotion Cope With Damage-Induced Neural Asymmetry?

**DOI:** 10.3389/fnsys.2022.828532

**Published:** 2022-03-03

**Authors:** Didier Le Ray, Mathias Guayasamin

**Affiliations:** Université de Bordeaux, CNRS, EPHE, INCIA, UMR 5287, Bordeaux, France

**Keywords:** sensory-motor integration, neuronal networks, injury, motor recovery, development

## Abstract

In most vertebrates, posture and locomotion are achieved by a biomechanical apparatus whose effectors are symmetrically positioned around the main body axis. Logically, motor commands to these effectors are intrinsically adapted to such anatomical symmetry, and the underlying sensory-motor neural networks are correspondingly arranged during central nervous system (CNS) development. However, many developmental and/or life accidents may alter such neural organization and acutely generate asymmetries in motor operation that are often at least partially compensated for over time. First, we briefly present the basic sensory-motor organization of posturo-locomotor networks in vertebrates. Next, we review some aspects of neural plasticity that is implemented in response to unilateral central injury or asymmetrical sensory deprivation in order to substantially restore symmetry in the control of posturo-locomotor functions. Data are finally discussed in the context of CNS structure-function relationship.

## Introduction

The development of pluricellular organisms depends primarily on the establishment of both radial and bilateral symmetries, which are considered to be determined genetically (Holló, 2015), even though symmetry breaks are necessary for certain physiological functions to be effective (Moubayidin and Østergaard, 2015). Nevertheless, body asymmetry is very common in invertebrates and ancestral vertebrates, whereas it is much less obvious in mammals even if they retain some of such characteristics (Andrew, 2009).

Asymmetries exist in the organization of all inner organs, including the central nervous system (CNS; Concha and Wilson, 2001; Blum and Ott, 2018; Schweickert et al., 2018). However, imaging approaches in human subjects indicated that no gross morphological nor functional asymmetry exist in the posturo-locomotor nervous system, from the cortex to the lumbar spinal cord (White et al., 1997), including cerebellar (Grodd et al., 2001) and cortical (Overduin and Servos, 2008) sensory-motor representation networks. Indeed, in vertebrates both locomotion and posture require bilateral symmetry in spinal sensory-motor circuits that mirrors the symmetrical organization of their biomechanical apparatus (Farel and McIlwain, 2000; Cooke, 2004). Such sensory-motor CNS symmetry, built during early development and refined during growth, has been shown to ensure symmetrical locomotion and effective postural adjustments in humans (Gervasio et al., 2015).

Much evidence from both human and animal studies indicates that the CNS is capable of considerable plasticity, both at the structural and functional levels. Such a plastic ability is evoked as soon as early development and is conserved into adulthood. The posturo-locomotor nervous system is also subject to significant plasticity in response to learning or accidental injury. For instance, although basically symmetrical, postural control in humans can be trained to become asymmetrical, expressed as a constant displacement of the center of pressure relative to the vertical body weight vector (Shiller et al., 2017), demonstrating the inherent ability of the symmetrically organized motor nervous system to generate a permanent asymmetrical command. Similarly, decerebrate cats walking on a laterally tilted treadmill are able to maintain body balance by transforming a naturally symmetrical locomotor pattern into an asymmetrical one (Musienko et al., 2014), and the enforcement of an asymmetrical gait to spinalized cats changes spinal sensory-motor processes to prevent additional disequilibrium (Hurteau and Frigon, 2018). In opposite, a sensory imbalance experimentally generated in the neck of squirrel monkeys induced a motor disequilibrium that was reversed within days (Igarashi et al., 1969). This latter suggests that in response to an imposed asymmetry (for instance, consecutive to central or sensory injury or disease) the posturo-locomotor neural system has the capacity to adapt and to restore symmetrical motor functions.

Here, we will review some of the neural mechanisms involved in restoring functional symmetry in the control of posture and locomotion in vertebrates, with a particular emphasis on vestibular unilateral deprivation. We will start with a brief reminder of the general organization of the neural posturo-locomotor system, followed by its structural and functional adaptations to imposed central or sensory asymmetry in both adults and subjects in development. We will particularly focus on research allowing to investigate the relationship between neural structure and behavioral function and how it is established during development.

## Posturo-Locomotor Networks in Vertebrates

### Spinal Networks

**FIGURE 1:**
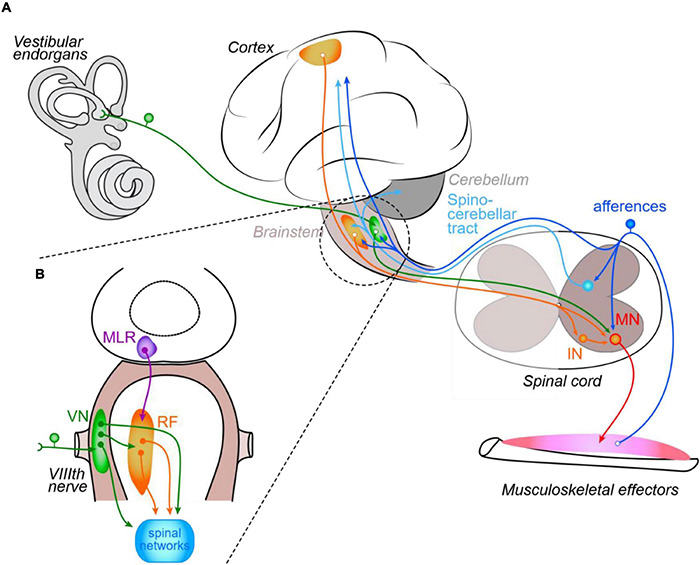
Schematic organization of the control of posture and locomotion in vertebrates. Illustrated central and peripheral structures and connecting pathways **(A)** are arbitrarily limited to those discussed in the review. The inset **(B)** focuses on the main brainstem structures responsible for posturo-locomotor supraspinal commands. IN, interneuron; MN, motoneuron; MLR, mesencephalic locomotor region; VN, vestibular nuclei; RF, reticular formation; VIIIth nerve, vestibular nerve.

The control of posture and locomotion requires the sequential and, at least in the case of locomotion, rhythmic activation of series of skeletal muscles bilaterally distributed along the body. Recruitment of these muscles during locomotion is primarily ensured by basic motor commands generated by the so-called central pattern generators (CPGs; Figure 1A; for recent reviews, see Côté et al., 2018; Grillner and Kozlov, 2021). Briefly, basic commands are organized by sets of excitatory interneurons (INs), which are arranged in modules generating fundamental recurring activity, and which are coordinated through inhibitions implicating a variety of local inhibitory INs (Cangiano and Grillner, 2005; Grillner et al., 2005; Berkowitz et al., 2010; Bagnall and McLean, 2014; Jay and McLean, 2021). Bilateral coordination depends specifically on commissural INs while ipsilateral (e.g., flexor/extensor) coordination depends on ipsilateral INs. These interneuronal networks connect motor neurons (MNs) that transmit central motor commands to effector muscles. However, MNs are not just passive output neurons. As it is the case for INs in the CPG, MNs possess some particular membrane properties, such as the persistent sodium current (I_*NaP*_; Tazerart et al., 2007, 2008), allowing them to integrate rather than just follow the numerous synaptic inputs they receive. Locomotor CPGs are localized in the spinal cord, and their organization depends on the animal biomechanical apparatus.

In fish or larval amphibian, both propulsion and posture are ensured by axial muscles that are organized in myotomes distributed along the body axis (D’Elia and Dasen, 2018) and controlled by bilaterally alternating neuronal networks, segmentally iterated throughout the spinal cord (Roberts et al., 2010; Berg et al., 2018). In contrast, in quadrupeds the CPGs dedicated to fore- and hindlimb movements are respectively segregated in cervical and lumbar segments (e.g., in rats: Ballion et al., 2001, and Cazalets et al., 1995, respectively), coordinated through propriospinal pathways (Juvin et al., 2005, 2012), and are putatively organized in interconnected modules, each responsible for the command of flexion/extension cycles at a single joint (Grillner and El Manira, 2020; Grillner and Kozlov, 2021). However, the exact nature and organization of CPGs in mammals remain unclear, although the rise of genetic approaches has already allowed the identification of several populations of INs putatively involved in rhythm-generating GPGs (Goulding, 2009; Rancic and Gosgnach, 2021).

The postural nervous system, in contrast, is largely distributed along the spinal cord. Although variations exist between species and in accordance with the type of posturo-locomotor movements they produce, each spinal segment participates in organizing the axial muscle contractions responsible for postural activities (reviewed in Guillaud et al., 2020). Globally, postural control can be separated into two major functions: one is dedicated to body stabilization in the absence of goal-directed movement and resists external perturbations including gravity, while the other is dynamically engaged during self-motion and ensures body balance and orientation in space. Usually, the control of posture, whether it concerns perturbation-induced reflexes or gait-related control, is considered to depend mainly on descending commands from supraspinal centers (reviewed in Takakusaki, 2017), partly because the available knowledge about the organization and dynamic activity of spinal postural networks is scarce. In fact, most data come from analyses of thoracic back muscles during locomotion. In rats (Falgairolle and Cazalets, 2007) as in humans (de Sèze et al., 2008), metachronal propagations of back muscle contraction were observed to occur with comparable patterns during walking. Moreover, such metachronal waves were found to persist in thoracic ventral root recordings during pharmacologically induced fictive locomotion in the *in vitro* isolated spinal cord of newborn rats, suggesting the existence of a propriospinal coordination between the CPG for locomotion and the thoracic networks (Falgairolle and Cazalets, 2007). However, similarly to fishes it remains quite impossible to ascertain that such back muscle activities are not also simply related to propulsion, which is made possible by the vertebral spine flexibility observed in the majority of vertebrate species. For example, during cheetah or greyhound run, cycles of spine curvature/extension strongly participate in the animal’s propulsion (see Hudson et al., 2012).

Nonetheless, the particular anatomy of the vertebral column of post-metamorphic anuran *Xenopus laevis* (Beyeler et al., 2008) strongly limits spine flexibility. Indeed, propulsion is entirely provided by the hindlimbs, whereas the contraction of the axial back muscles *dorsalis trunci* generates only small twists of one vertebra with respect to the neighboring others, conferring these muscles a purely postural function (at least for myomeres 3 and 4 since their insertion on the skeleton prohibits any participation in propulsion; Olechowski-Bessaguet et al., 2020). Electrophysiological recordings from either back muscles *in vivo* or *dorsalis*-innervating thoracic ventral roots *in vitro* showed strict coordination with hindlimb muscles/lumbar ventral roots activity during swimming, and thoracic MNs were likely directly activated by the lumbar CPG (Beyeler et al., 2008). Thus, in juvenile *Xenopus* at least, axial postural networks are subdued to propulsive networks through segmental propriospinal connections when the locomotor CPG is active, in order to generate the appropriate anticipatory postural adjustments necessary to ensure accurate locomotion.

### Sensory and Supra-Spinal Control of Spinal Networks

Although intrinsic auto-organization of posturo-locomotor activities exists within spinal networks, these are modulated by both local sensory feedbacks and descending commands from supra-spinal structures (Figure 1; Grillner and El Manira, 2020). During locomotion, local sensory control is mediated by segmental proprioceptive and cutaneous afferents in a cycle-dependent manner (MacKinnon, 2018). For instance, load-mediating afferents (Ib) are implicated in joint extension while afferents that inform about muscle length variation (Ia afferents from muscle spindles) are mainly involved in joint movement termination and initiation of movement in the opposite direction, acting on the reciprocal inhibition between flexor and extensor (e.g., in humans: Perez et al., 2003). Cutaneous information is involved equally in locomotor and postural activities; notably during locomotion, they modulate the cycle by interacting presynaptically with other afferent signals. In turn, during locomotion sensory feedback afferents are cyclically modulated by the CPG via specialized inhibitory INs (reviewed in Côté et al., 2018). Descending commands from supra-spinal centers are also able to modulate spinal reflexes in a phase-dependent manner. In humans for example, increasing the postural threat during walking changes the gain of the H-reflex (Krauss and Misiaszek, 2007), which suggests that convergence of both local sensory and descending brain commands onto spinal INs (Stecina and Jankowska, 2007; Stecina et al., 2008) is essential to regulate local reflexes (Phadke et al., 2009). Nevertheless, even in spinalized cats (deprived of every supra-spinal inputs) walking on a treadmill, pace can gain symmetry if treadmill velocity is increased (Dambreville et al., 2015), indicating that spinal networks are nevertheless intrinsically able to adapt the motor program they produce as gait increases in speed, and this probably via the implication of the local, symmetrically organized sensory feedback networks.

Supra-spinal structures determine initiation and termination of locomotion, as well as gait adaptation to challenging milieus, in a cascade of commands sequentially involving cortical and thalamic structures, basal ganglia, mesencephalic nuclei and, finally, reticulospinal nuclei that directly control spinal CPGs (Grillner et al., 2008; Kozlov et al., 2009; Leiras et al., 2022). They are also responsible for a majority of postural commands, besides the propriospinal coordinating pathways involved in locomotion-required postural adjustments (see above). Among these structures, sensory-motor cortices, via bilateral crossed corticospinal tracts, are mostly implicated in the voluntary control of fine body and limb motion (see Takakusaki, 2017). In animals in which the cortex is much less developed, the fine control of movement seems to be achieved by the red nucleus (Olivares-Moreno et al., 2021), which is intimately interconnected with both the cortex and the cerebellum (Cacciola et al., 2019). Basal ganglia also participate in posture regulation, through projections on brainstem motor centers, including the mesencephalic locomotor region (MLR) and the reticular formation (Takakusaki et al., 2016). Within the brainstem (Figure 1B), vestibulospinal (VS) and reticulospinal (RS) nuclei play the major role, reticulospinal neurons relaying the great majority of higher posturo-locomotor commands from the MLR (as described in many details in the lamprey; see Le Ray et al., 2011) and cortex (Stecina and Jankowska, 2007) as well as inputs from the central vestibular nuclei (CVNs; e.g., Deliagina et al., 1992; Pflieger and Dubuc, 2004). Yet, in all vertebrates vestibulospinal neurons also directly activate spinal motor networks to ensure body postural adjustments and maintain head position in space (Straka and Baker, 2013; Cullen, 2016; Bagnall and Schoppik, 2018). In addition, CVN neurons affect indirectly the control of posture via their projections to the thalamus and insular cortex, as well as via their bilateral interactions with cerebellar nuclei (see Takakusaki, 2017; Cullen, 2019). The latter also directly and indirectly integrate vestibular afferents (Carpenter et al., 1972; Schniepp et al., 2017).

Many studies investigated the organization of vestibulospinal projections in various species, from lamprey (Deliagina et al., 2014) to mammals (Kasumacic et al., 2015); however, only a recent work in the juvenile *Xenopus* clearly provided a functional and anatomical description of vestibulospinal projections onto spinal neurons specifically involved in the control of posture (Olechowski-Bessaguet et al., 2020). This study reports that vestibulospinal nuclei activate identified postural MNs through two distinct ways: the first, classically described in all species, consists of a direct activation of populations of spinal neurons (INs and MNs) involved in the control of posture at the segmental level (e.g., thoracic MNs activating *dorsalis* muscles), and conveys principally signals related to head position; the second, yet undescribed, consists of an indirect pathway to activate these same thoracic neurons via a lumbar interneuronal relay dispatching the vestibular command to thoracic and lumbar MNs simultaneously, and conveys mainly velocity signals from the vestibular system.

Whatever the species, central vestibular neurons integrate spatially corresponding, convergent vestibular, optokinetic, proprioceptive and cutaneous inputs, and it has been postulated that the vestibulospinal network anatomical organization included all sensory-motor transformations appropriate for the control of posture (Vidal et al., 1993). Interestingly, although working according to a “push-pull” mechanism (one side being inhibited when the other side is excited by a given direction of head movement) the vestibular system is symmetrically organized, and mathematical models have suggested that the bilateral symmetry of vestibular reflexes (notably the vestibulo-ocular reflex; Smith and Galiana, 1991) resulted from this symmetry in the vestibulo-motor system (e.g., Ris et al., 1995). In particular, vestibulospinal projections are anatomically organized in symmetry groups likely related to their physiological sensory-motor function (McCollum, 2007), providing the vestibulospinal system with a powerful capacity to adapt precisely spinal posturo-locomotor functions. However, similarly to segmental sensory feedbacks in the spinal cord, vestibular integration is subject to modulation during active movement (Chagnaud et al., 2015), notably via interactions with the cerebellum (Takahashi and Shinoda, 2021) and via bilateral ascending spino-bulbar pathways conveying an efference copy of the motor program generated by spinal networks. These latter ascending efferent copies were clearly demonstrated in both larval (Combes et al., 2008) and juvenile (von Uckermann et al., 2013) *Xenopus* to coordinate directly eye movements with locomotor-induced head displacements, and were proposed to exert side-specific filtering of vestibular sensory integration in the CVNs (Straka et al., 2018). Recent studies in human subjects provided evidence that comparable efference copy mechanisms may also account for the stabilization of eye vertical position, at least during fast locomotion (Dietrich and Wuehr, 2019; Dietrich et al., 2020).

## Compensation After a Unilateral Lesion in Central Motor Circuits

The anatomical and functional organization of the sensory-motor neural system involved in posture and locomotion is globally symmetrical, both at spinal and supra-spinal levels, fitting the gross symmetry of the biomechanical apparatus that ensures these functions. It is known that any loss in symmetry in either the neural or biomechanical arrangement would generate discrepancies between the two systems. Amputation or immobilization of a limb, for instance, will cause strong reorganization in various parts of the neural system (Bramati et al., 2019; Makin and Flor, 2020; Conboy et al., 2021; Raffin, 2021), while an accidental or pathological neural asymmetry will trigger plasticity in both the neural and biomechanical (e.g., muscle; Gorgey et al., 2019) systems. In the following, we will only review studies regarding neural plasticity and behavioral adaptation in response to damage-induced central asymmetry. One difficulty in understanding how the posturo-locomotor CNS copes with accidental central asymmetry resides in the fact that, depending on animal models and location of the lesion, various levels of recovery can be observed. For instance, fish and most amphibians are able to restore a substantial part of transected axons within the spinal cord (Noorimotlagh et al., 2017; Rasmussen and Sagasti, 2017; El-Daher and Becker, 2020), making these animals particular models of posturo-locomotor symmetry restoration that will not be considered in detail in the following. In addition, for comparable lesions in a given animal model, the motor activity generated below the lesion also largely depends on the internal state of excitability of the considered spinal networks and local sensory feedback, as well as of spared descending commands. Nevertheless, some general rules can be drawn from the vast literature in this domain.

### Structural Brain Plasticity After Unilateral Central Nervous System Lesions

**FIGURE 2:**
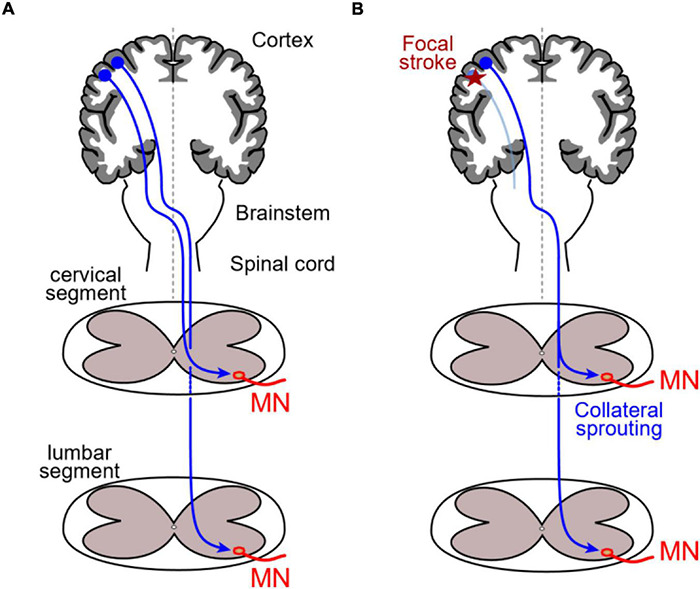
Unilateral stroke-evoked corticospinal plasticity. **(A)** In normal rats, corticospinal neurons from sensory-motor areas (dark blue projections) specifically connect either cervical or lumbar spinal MNs respectively involved in fore- and hindlimb fine motricity. **(B)** After a focal stroke in the cortical region hosting neurons projecting into cervical segments (red star), forelimb-related corticospinal neurons degenerate (light blue), and hindlimb-related neurons normally projecting only onto lumbar MNs sprout collaterals that make synapse onto cervical MNs deprived of their normal corticospinal inputs.

It has been long known that damages in the human sensory-motor cortex produce side-specific effects (Robinson, 1979), with acute lesions on the right side affecting mostly the control of posture while left side brain injuries rather generate apraxia (Spinazzola et al., 2003). As a primary response to unilateral stroke in the motor cortex, deep cortical reorganization occurs, as recently reported in patients with focal ischemic stroke where theta burst-induced depression was initially higher in the contralesional motor hemisphere and slowly recovered during the following weeks, whereas no changes were observed in the ipsilesional hemisphere (Hordacre et al., 2021). In rodents, stroke on one side of the sensory-motor cortex similarly triggers contralateral motor cortex plasticity (Takatsuru et al., 2009), but may as well affect the ipsilesional cortex. As shown in the adult rat, focal ischemic stroke in the motor cortex area controlling a forelimb initially generates motor deficits that are progressively compensated for, due to ipsilesional motor cortex neurons involved in hindlimb control sprouting new collaterals into cervical spinal motor circuitry (Figure 2; Starkey et al., 2012).

In contrast, a developmental alteration of the cerebellum symmetrical organization (unilateral hypoplasia) does not significantly modify postural and locomotor movement symmetry (Immisch et al., 2003) but globally slows down movements and delays motor skill acquisition in children (Benbir et al., 2011), suggesting that cerebellar symmetry is not a pre-requisite for accurate motor control. However, it has been shown in primates that, depending on the considered cerebellar network, a unilateral acute lesion oppositely affects nystagmus direction (Romano et al., 2020), suggesting that posture and eye movement rely on distinct neural organizations. In rats, posture is comparably affected by acute lesions of the sensory-motor cortex on either side; however, the reported asymmetry in hindlimb postural response seems to rely on distinct mechanisms, a right-side damage triggering spinal motor network reorganization, and a left-side lesion instead altering local sensory integration (Zhang et al., 2020).

As a result of spinal cord injury (SCI), disconnection of descending pathways that carry supra-spinal motor commands toward spinal motor networks generates postural and locomotor disturbances (e.g., reduced use of ipsilesional limb after unilateral rubrospinal tract lesion; Webb and Muir, 2003) that can be (partially) compensated for by downstream (see later) and/or upstream plasticity. Such plasticity can occur spontaneously but usually benefits from sensory-motor training similarly in patients and animals (Knikou, 2010; Torres-Espín et al., 2018; but see Fouad et al., 2000). In rats, SCI does not trigger damaged corticospinal neurons to degenerate (Nielson et al., 2010) but these are likely redeployed in new functional cortical networks. For instance, incomplete SCI at a cervical level leads to a transient loss of ipsilateral paw representation in the primary somatosensory cortex; this representation is reactivated (Ghosh et al., 2009; Martinez et al., 2010; Bazley et al., 2014) and tactile ability ameliorates with sensory-motor training protocols, mobilizing the preserved pathways and resulting in primary motor cortex reorganization (Martinez et al., 2009, 2010). On the contralesional side, consecutive to cord hemisection and bilateral dorsal column cut, motor cortex spiny pyramidal neurons exhibit tapered and longer dendrites, due to an overexpression of polysialylate cell adhesion molecules that limit synapse formation (Kim et al., 2006, 2008), indicating cellular reorganization in cortical networks. Finally, plasticity may occur as well in the lower brainstem, as recently investigated in lampreys (Hough et al., 2021) where spinal cord hemisection leads ipsilesional RS neurons to adapt their electrical properties, compared to contralateral uninjured RS neurons. However, such changes in intrinsic properties do not seem to affect the way these command neurons integrate input signals, notably sensory trigeminal inputs, raising the question of the functionality of such a plasticity and whether this participates in subsequent locomotor recovery.

### Functional Spinal Plasticity After Unilateral Spinal Cord Injury

Spinal cord injured animals are common models for studying neural plasticity (Dietz and Fouad, 2014; Alizadeh et al., 2019) in the context of motor functions (respiration: Streeter et al., 2020; posture and locomotion: Brown and Martinez, 2019). Most species are capable of partial or complete recovery after SCI, demonstrating the intrinsic ability of spinal sensory-motor networks to organize functionally relevant motor commands even in the absence of part or all descending commands and neuromodulation (Rossignol et al., 1996; but see also Merlet et al., 2021). Lumbar SCIs are more deleterious than mid-thoracic ones as reported in cats (Rossignol et al., 2002) and rats (García-Alías et al., 2006), probably because they may directly injure the locomotor CPG. In this context, spinal cord hemisection or incomplete SCI at a cervical or thoracic level provide interesting models to investigate the mechanisms by which a bilateral symmetrically built CNS adapts to abnormal imbalance, in order to maintain symmetrical biomechanics as accurate as possible. For instance, whereas a cervical cord hemisection induces the operational loss of the ipsilesional forelimb in rats, both hindlimbs recover coordinated activity compatible with functional locomotion (Ghosh et al., 2009).

In cats with unilateral thoracic SCI, after intensive motor training both the spinal CPG and sensory-motor connections exhibit deep remodeling (Barrière et al., 2008; Martinez et al., 2013), which results in the generation of motor command asymmetries downstream of the lesion to restore bilateral locomotion. In addition, such reorganized spinal networks persisted after a subsequent complete spinal cord transection, the latter transiently triggering opposite asymmetries before normalization (Barrière et al., 2008, 2010), which demonstrated that initial symmetry restoration was independent from descending influences. In contrast, other studies in animals (Singh et al., 2011) and humans (Edgerton et al., 2001) tend to suggest that recovery from unilateral SCI depends strictly on an interplay between contralesional descending pathways and local sensory feedback to enhance depolarization in spinal neurons below the lesion at least primarily; thereafter, when spinal circuits retrieve their ability to respond to descending commands without additional excitation, reflexes would be down-regulated (Little et al., 1999). Experiments in cats showed that restoration substantially relied on the asymmetrical integration of local reflexes, notably cutaneous-mediated reflexes (Helgren and Goldberger, 1993; Frigon et al., 2009) that were enhanced in the ipsilesional lumbar hemicord (Gossard et al., 2015). Similar implication of cutaneous inputs was also reported from thoracic hemisection experiments in the chick (Muir and Steeves, 1995; Muir et al., 1998), and conditioning the control of H-reflex amplitude on the ipsilateral side reduces motor asymmetry in rats, monkeys and humans (Wolpaw and Lee, 1989; Chen et al., 2006; Thompson and Wolpaw, 2015). In contrast, prior ankle denervation prevented symmetrical posturo-locomotor recovery in SCI cats (Carrier et al., 1997). All these results support a pivotal role for local sensory integration in the recovery of functional motor symmetry. Furthermore, mutant mice lacking proprioceptive feedback from muscles showed no recovery after a thoracic hemisection, and this was associated with a restricted and inaccurate reorganization of contralesional descending pathways below the lesion (Takeoka et al., 2014). This latter study thus suggests that if an interplay between descending pathways and local sensory feedback is required for the accurate reorganization of spinal networks after unilateral SCI, local sensory information likely orchestrates this interplay.

Plasticity is not limited to the restoration of hindlimb CPG operation. After a cervical hemisection, modifications were found in the unaffected forelimb circuitry that compensated for the motor deficits in the harmed forelimb in adult rats (Khaing et al., 2012), and the bilateral propriospinal coordination between cervical and lumbar networks (respectively controlling forelimbs and hindlimbs) is changed in order to bilaterally restore the anteroposterior interlimb coordination in cats (Côté et al., 2012). Although significant posturo-locomotor recovery occurs, motor anomalies may nevertheless persist after incomplete SCI and subsequent training. In adult rats, for instance, persistent overlap between flexor and extensor muscle activities was reported and proposed to result from the existence of an asymmetry in descending pathways (Kaegi et al., 2001). However, the authors also reported a simultaneous improvement of stance and body support in lesioned animals, which suggested that what appeared to be an incomplete recovery might in fact consist of a compensatory motor adaptation to the loss of half of the descending motor commands. In fact, global reorganization of muscle activation pattern was similarly described where axial muscles below the lesion were found to participate more during pelvis and hindlimb movements in trained SCI rats (Giszter et al., 1998), as well as during phase shift in walking patients (Scivoletto et al., 2007). Such axial muscle plasticity further raises the question of why axial networks seem to be less affected by unilateral central lesions. An explanation may be found in the particular organization of descending commands onto these networks where pyramidal excitatory commands onto dorsal MNs are mainly relayed by RS neurons and inhibitory commands by spinal inhibitory INs located in the two hemicords. Such bilaterally distributed organization may account for the axial system greater resilience to unilateral pyramidectomy in cats (Galea et al., 2010).

### Unilateral Spinal Cord Injury-Induced Spinal Pathway Plasticity

**FIGURE 3:**
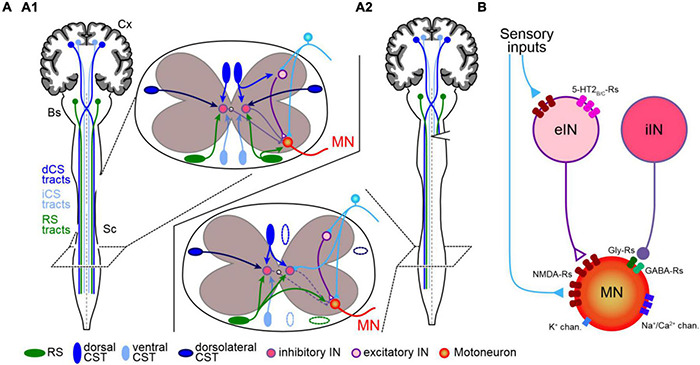
Spinal network reorganization after unilateral spinal cord injury. **(A)** The symmetrical organization of uncrossed (light blue) and crossed (dark blue) corticospinal and reticulospinal (green) projections (illustrated in upper inset) and local sensory inputs (cyan) onto segmental INs (pink) and MN (orange) in control animal **(A1)** exhibit adaptations below spinal cord hemisection **(A2)** ipsilesional sensory inputs invade IN and MN dendritic territories deprived from their ipsilateral descending projections, and contralateral descending axons sprout terminals into the ipsilesional hemicord. See text for details. Cx, cortex; Bs, brainstem; Sc, spinal cord; iCS and dCS, ipsilateral and decussating corticospinal tracts; RS, reticulospinal. **(B)** SCI-induced acute modifications in motor neuron (MN) and excitatory and inhibitory interneurons (eIN and iIN, respectively) intrinsic and synaptic properties leading to MN hyper-excitability. Sensory-evoked responses are enhanced due to increased expression of NMDA receptors (NMDA-Rs) in both MN and eIN, and increased expression of serotonin receptors (5-HT2_*B/C*_-Rs) in eIN. In contrast, inhibitory influences from iIN onto MN are reduced due to lower motoneuronal expression of GABA and glycine receptors (GABA-Rs and Gly-Rs, respectively). In addition, overexpression of sodium/calcium permeant channels (Na^+^/Ca^2+^ chan.) and lower expression of potassium channels (K^+^ chan.) intrinsically increase MN excitability. The relative expression of membrane channels and receptors is illustrated as follows: one item shows a decrease and three items an increase compared to a “normal expression” of two items.

Because both ascending (principally sensory) and descending (motor) pathways are affected by SCI protocols, series of experiments with restrained spinal lesion were designed with the aim of clarifying the relative participation of different pathways in SCI acute effects and subsequent recovery. Hence, a joint unilateral destruction of dorsolateral funiculus (corticospinal and reticulospinal tracts) and ipsilateral dorsal columns (ascending sensory tract) had no long-lasting impact on posture and locomotion in adult rats. In contrast, it produced strong deleterious effects when combined with ventrolateral funiculus (principal reticulospinal tract) interruption (Loy et al., 2002), confirming the major role of RS neurons in the control of downstream motor networks. Such a role was further supported by the observation in rats that post-SCI locomotor recovery necessitated the preservation of at least part of spinal ventral and/or lateral white matter (Schucht et al., 2002). Furthermore, increased crossing of contralesional RS fibers and increased number of connections on local propriospinal INs were reported to develop below the hemisection site, together with increased responses of these RS neurons to stimulation of the contralesional MLR (Rangasamy, 2013; Zörner et al., 2014; Engmann et al., 2020). This suggests that plasticity in brainstem motor command circuitry occurs to compensate the functional loss of a half of command neurons while spared RS neuron projections reorganize to restore functional symmetry in projection pathways below the lesion (Figure 3A).

In contrast, after unilateral spinal hemisection no significant changes were globally found in cortico- and vestibulospinal tracts, and only a small tendency of increased rubrospinal fiber crossing was reported (Zörner et al., 2014). However, selective spinal lesion targeting the decussating corticospinal tract on one side induced motor deficits that recovered rapidly in rats (Bazley et al., 2012). This was paralleled by the induction of new connections between corticospinal terminals and long-projecting propriospinal INs that connected lumbar MNs (Bareyre et al., 2004), the outgrowth of ipsilaterally projecting corticospinal fibers into sub-lesion cord segments, as well as sprouting of ipsilesional sensory afferents within the ventral cord territories that lost their corticospinal inputs (Figure 3A; Bareyre et al., 2002; Brus-Ramer et al., 2007; Tan et al., 2012). The establishment of new corticospinal connections and subsequent motor recovery are prevented in mutant mice expressing inhibitory DREADDs in spinal INs, demonstrating that this process depends on the presence of activity in local INs, likely induced by segmental sensory afferent activation of NMDA receptors (Bradley et al., 2019). Consecutive to sensory-motor cortex unilateral stroke (which causes corticospinal neuron degeneration) hindlimb stretch reflexes were found to be enhanced and some plasticity genes (e.g., Grin2a/DLg4 and Tgfb1) inversely regulated in adult rat lumbar segments (Zhang et al., 2020). This study suggests that one important function for sensory-motor cortex neurons projecting to the spinal cord is to regulate plastic adaptation of local spinal reflexes during ongoing posturo-locomotor activity. Indeed, phase-dependent depression of reflex loops was found to be reduced following incomplete SCI and able to recover, in a neurotrophic factor-dependent manner (Côté et al., 2011), in response to spared corticospinal pathway stimulation (Brus-Ramer et al., 2007; Petrosyan et al., 2020) or sensory-motor training (Phadke et al., 2009; Côté et al., 2011). Corticospinal damage-induced spinal reorganization acutely leads to spinal hyperreflexia, which has been considered as maladaptive (Tan et al., 2012), though such hyperreflexia may participate actively in the process of posturo-locomotor recovery by increasing the overall excitability of spinal networks below the lesion site (see below).

Dorsal hemisection, which suppress half of sensory inputs ascending to brain centers, has dramatic acute effects on locomotion, affecting notably paw placing during skilled walking, until sensory afferent reorganization within the ipsilesional hemicord occurs and allows functional recovery. In mice with such lesions, ascending dorsal root neurons increased branching below the lesion site and, in particular, connected propriospinal INs projecting into the cuneate nucleus, constituting so a detour circuit to brainstem centers (Granier et al., 2020), and beyond, to the primary sensory-motor cortex (as afore suggested in rats and monkeys; Kaas et al., 2008). Functional restoration of bilateral tactile and proprioceptive sensation occurred in these animals within weeks, demonstrating the exceptional ability of sensory systems to compensate for selective alterations of their central pathways. In contrast, adaptation of central motor commands to unilateral sensory loss noticeably takes much longer to reorganize. Indeed, it was shown in macaque monkeys that a unilateral dorsal rhizotomy at the cervical level (which completely removed sensory inputs from one forelimb) triggered a complete loss of grasp in the corresponding forelimb that persisted over several months. With time, functional recovery nevertheless occurred and was paralleled by the anatomical reorganization of corticospinal projections into cervical segments from both the primary somatosensory and motor cortices (Darian-Smith et al., 2013; Nardone et al., 2013); however, while the former displayed clear retraction from the cervical hemicord, the latter exhibited abnormal sprouting in the ipsilesional dorsal horn (Darian-Smith et al., 2013; but see also Darian-Smith et al., 2014). These works demonstrate that a unilateral sensory deprivation causes rearrangement of different corticospinal projections, and further suggest a distinct implication of each corticospinal tract in motor recovery that remains unexplained.

### Cellular Mechanisms of Unilateral Spinal Cord Injury-Induced Spinal Network Plasticity

Whereas the effects of cord hemisection on directly lesioned tissues are well documented (Ahuja et al., 2017), the cellular impacts on downstream motor networks in relation with the observed behavioral consequences is still not clear, and some aspects have not been explored to date. For example, the astrocyte role in SCI-induced formation of glial scars (astrocytic reaction) has been analyzed extensively at the lesion level itself (Okada et al., 2018). Surprisingly, even though they are known to regulate activity of locomotor networks in physiological conditions (e.g., Mu et al., 2019) the potential implication of astrocytes in post-SCI motor adaptation remains undocumented. In contrast, numerous investigations in various species have analyzed the neuronal mechanisms involved in motor restoration. In injured lampreys, spinal neurons below the lesion exhibited increased resting membrane potential as well as increased input resistance that, combined with synaptic efficacy enhancement, strongly improved neuronal excitability (Cooke and Parker, 2009). In adult rats with cervical cord hemisection, Gonzalez-Rothi and colleagues reported the transient development of muscular atrophy in the corresponding forelimb (Gonzalez-Rothi et al., 2016), which could have suggested a deficit in motor command due to dramatic and maladaptive rearrangement of spinal motor and/or premotor neurons. But, using pseudorabies viruses during the same period of time in order to track putative added/subtracted connections, the authors did not observe any obvious changes in first-order premotor IN-MN circuitry of the cervical cord (Gonzalez-Rothi et al., 2015), suggesting that no dramatic premotor network reorganization occurred that could explain muscle atrophy-related reduced motoneuronal activity, nor its recovery with time. Furthermore, it was shown in mice that genetically identified V2a (Shox2) INs, which are putative constituents of the lumbar CPG, conserved their intrinsic resting membrane properties after SCI (Husch et al., 2012). However, these INs exhibited increased excitability due to enhanced efficacy of both local sensory and descending monoaminergic input synapses, as well as more intense expression and increased sensitivity of 5-HT_2*B*_/_2*C*_ receptors (Figure 3B; Husch et al., 2012; Garcia-Ramirez et al., 2021). Noteworthy, these serotonin receptors facilitate synaptic input-evoked responses of spinal neurons, likely by modulating calcium channels sustaining plateau potentials (Perrier et al., 2013). Taken together, it seems that spinal INs are only minimally affected by spinal hemisection, suggesting a subsequent minor role in motor symmetry restoration. Nevertheless, because these INs integrate inputs from both local sensory afferents and descending projections from brainstem command centers, which both exhibit deep adaptation (see above), it is likely that plasticity at IN input synapses would allow more efficient network operation. Hence, increasing inhibition from commissural INs would participate in the restoration of bilateral coordination, while enhanced ipsilateral inhibition would participate in reducing MN hyper-excitability and in shaping new adapted coordination.

In contrast, diverse modifications were reported in MNs in rodents which underwent SCI (Figure 3B). Sub-lesion MNs were characterized by increased expression of sustained Na/Ca-dependent plateau potentials (Li et al., 2004; Heckman et al., 2005) and NMDA receptors (Gransee et al., 2017), both increasing MN responses to excitatory synaptic inputs. Moreover, structural alterations of motoneuronal input synapses favoring MN excitability were reported in the lamprey (Cooke and Parker, 2009), and a reduction of potassium conductances occurs in ipsilesional leg MNs after unilateral interruption of the corticospinal tract in human patients (Jankelowitz et al., 2007). Rodent sub-lesional MNs were also shown to express less chloride transporters KCC2 at their plasma membrane, which diminishes the impact of the inhibitory inputs they integrate (Boulenguez et al., 2010). Moreover, SCI triggered a decreased expression of membrane receptors to inhibitory neurotransmitters GABA and glycine in downstream MNs (Sadlaoud et al., 2010, 2020), which modifies motoneuronal integration of inhibitory inputs (de Leon et al., 1999). Globally, this combination of compensatory changes in motoneuronal properties results in a dramatic increase in MN excitability. One may ask whether changes in MN dendritic morphology could happen after a cord hemisection, since modified dendritic arbors were reported in lumbar MNs from rats with complete thoracic transection (Gazula et al., 2004). Noteworthy, phrenic MNs do not exhibit morphology changes after a cervical hemisection, whereas it is the case after complete activity blockade with tetrodotoxin (Mantilla et al., 2018). Thus, dendritic morphology likely is directly linked to overall activity rather than to precise input synapses. As a consequence, because cord hemisection globally increases neuronal activity we may not expect significant dendritic morphology changes in sub-lesion MNs. Interestingly, it has been reported from mice that acute thoracic hemisection reduced the number of input synapses onto MNs located upstream of the lesion (Goldshmit et al., 2008), an effect that was reversed by motor training. This latter result demonstrates that MNs throughout the spinal cord are subject to plasticity in response to localized SCI and further support the idea that global symmetry restoration in posturo-locomotor functions likely depends on cellular modifications spreading all over central motor circuits.

Either the large majority of studies until now have focused on MN properties and very few on IN ones (yet, see the demonstration of IN neurotransmitter switching in the autonomic system after spinal lesion; Hou et al., 2016) or the principal cellular target of unilateral SCI-triggered plasticity is the MN. This gap in knowledge questions the inhibitory network modification that has been proposed to account for the alteration of sensory integration in humans with incomplete SCI (Knikou, 2012), which could finally result also/instead from changes in motoneuronal integrative processes below the lesion as shown in adult rats (Sadlaoud et al., 2020). Further investigations at the cellular level will be necessary to unravel the relative participation of network INs and output MNs in motor recovery after unilateral SCI.

## Compensation After Unilateral Sensory Lesion, With an Emphasis on the Unilateral Loss of Vestibular Inputs

Altering sensory integrity induces the functional and anatomical reorganization of cortical maps (Buonomano and Merzenich, 1998; Power and Schlaggar, 2017) as well as functional adaptations in motor circuits both at the spinal and supra-spinal levels (Rossignol, 2006), which allows the restauration of postural and locomotor controls. For example, hindlimb deafferentation in cat acutely causes severe motor deficits that are largely compensated for with time. However, whereas the basic locomotor program generated by spinal CPGs is rapidly improved, step accuracy recovery requires several additional days and involves the joint implication of sensory afferents from the thoracic postural sensory-motor system (Goldberger, 1977) and various motor commands descending from the brain. This gave rise to the initial idea that motor adaptation to sensory deprivation resulted from ‘behavioral substitution’ rather than network remodeling (Goldberger, 1988). Supporting this hypothesis, the experimental deafferentation of one leg in healthy humans bilaterally modified leg biomechanics (Thoumie and Do, 1996) and bilaterally altered flexor and extensor muscle activation, resulting in postural adaptation by which the center of pressure was displaced backward under the ipsilesional foot (Imai et al., 2005).

There are also many studies which have identified configurational changes in various parts of the CNS following unilateral sensory deprivation. In that respect, a dorsal rhizotomy at cervical level C4–C8 in adult rats was shown to reduce specifically the representation of corresponding forelimb distal joints (mostly replaced by elbow representation) in the contralesional cortex, without changing the stimulation threshold to evoke left finger movements, nor affecting the cortical representation of any other limb (Jiang et al., 2013). Functional imaging in healthy subjects showed that acute deafferentation of a limb similarly induces cortical map reorganization. Indeed, an ischemic nerve block suppressing all distal afferent feedback from the left arm triggered a reversible expansion of the area in the left primary sensory-motor cortex that is activated during right finger movement and, as expected, the disappearance of responses to left finger stimulation (Kurabe et al., 2014). In the same way, in lamprey, crushing the trigeminal afferent nerve on one side induced a transient loss of touch-evoked escape response that was recovered due to partial anatomical restauration of the lesioned sensory fibers and return of bilaterally normal RS neuron responses (Calton et al., 1998). Likewise, crushing a sciatic nerve in newborn rats is followed by a motor reinnervation of leg muscles after several months. However, muscle activation remains definitively inaccurate, with permanent alteration of stretch reflexes (Alvarez et al., 2010) and aberrant activation of ipsilesional flexor muscles during quiet stance as well as during extensor phase when the animal walked (Vejsada et al., 1991). Because muscle spindles do not regenerate, it is likely that the absence of sensory feedback on the lesioned side prevented a complete adaptation to occur. Thus, it appears globally that central networks are more or less able to adapt to an asymmetrical loss of motor-related sensory inputs, although the underlying cellular mechanisms remain usually unraveled. Yet, the effects of a unilateral vestibular deprivation (UVD) and the subsequent cellular events that lead to motor recovery have been quite well described in many vertebrate species. The following of this chapter will focus on this particular sensory deprivation in which acute effects were clearly separated from chronic, compensated ones.

### Early Unilateral Vestibular Deprivation-Induced Modifications

#### Behavioral Consequences

Alterations of both static and dynamic vestibular reflexes are comparably observed immediately after unilateral vestibular neurectomy (suppressing also the Scarpa vestibular ganglion) or peripheral endorgan destruction (labyrinthectomy) in humans and animal models (Lacour et al., 2016; Simon et al., 2020). Static deficits affect both posture (head and body tilt toward the lesioned side), eye position (spontaneous nystagmus), as well as perception leading to vertigo in humans (tilt of vertical estimation). In the following, we will address only UVD-induced posturo-locomotor deficits and the related cellular mechanisms implicated during acute and chronic (compensated) phase. For complete information about vestibulo-ocular function and pathways and UVD-induced plasticity, see Straka and Dieringer (2004); Beraneck and Idoux (2012), and Cullen (2016).

Posturo-locomotor deficits were proposed to result from the loss of both otolith and semi-circular canal information (Mbongo et al., 2005). Yet, utricle selective ablation in terrestrial frogs reproduces postural deficits (Straka and Dieringer, 1995), whereas all motor deficits are observed in rats where only canal signals are impaired by the use of canal plugs (Geisler et al., 1997). Altogether, canal information may be the predominant signal for head stabilization in animals (including humans) whose head acquired additional mobility with respect to the thorax, while otolithic signals have prominence in the control of body vertical orientation in all species (Fritzsch et al., 2014). Whereas static deficits are rapidly compensated for (about a week post-lesion in animal models, up to 3 months in humans; Lacour et al., 2016), vestibular-related reflexes never totally recover, with the gain of vestibulo-ocular reflexes remaining lower than control, and postural adjustment enduring persistent deficits in challenging situations (Vidal et al., 1998).

#### Central Vestibular Alterations

**FIGURE 4:**
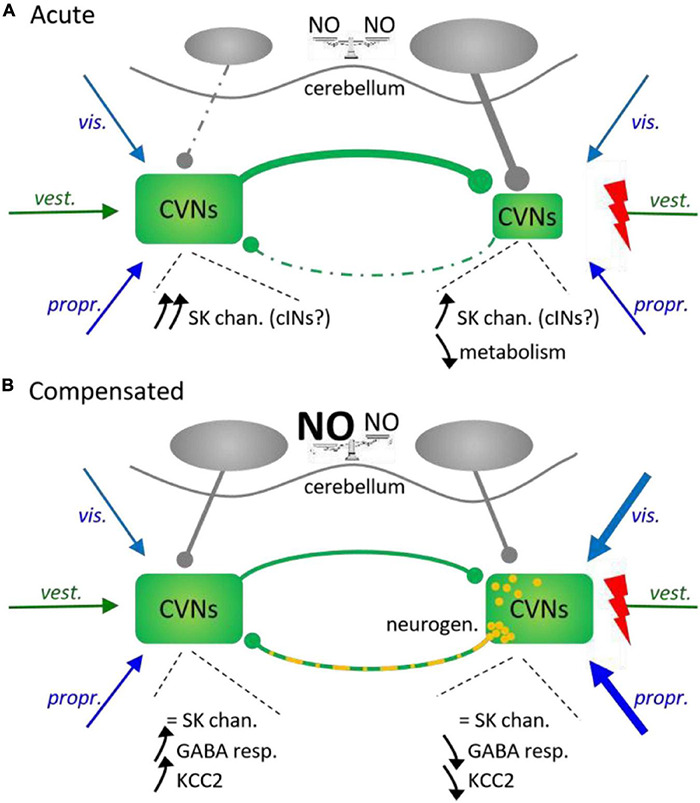
Unilateral vestibular deprivation-induced adaptations in central vestibular nuclei and cerebellum. **(A)** Acute effects. A unilateral loss of vestibular afferents (*vest.*) triggers a rapid decrease in ipsilesional CVN neurons activity due to conjoint intrinsic and metabolic modifications together with increased inhibition from contralateral central vestibular nuclei (CVNs) and ipsilateral cerebellar nuclei. Although intrinsic properties of CVN neurons change bilaterally, net effect consists of a disequilibrium in reciprocal inhibition through commissural vestibular pathways (cINs) resulting in a stronger inhibition of ipsilesional CVNs. *vis.*, *propr.*, visual and proprioceptive afferents; NO, nitric oxide. Dotted lines: underactivated influences. **(B)** Compensated cerebello-vestibular network. Restoration of bilateral balance results from additional changes in intrinsic and synaptic properties of CVN neurons on both sides, together with ipsilesional GABAergic neurogenesis (yellow dots) and increased synaptic weight of both visual and proprioceptive inputs onto ipsilesional CVNs. Bilateral balance restoration also occurs in cerebellar nuclei due to a relative increase in NO on the contralesional side.

Posturo-locomotor dysfunctions induced by a unilateral vestibular alteration result primarily from the disequilibrium that is generated by asymmetrical activity between ipsi- and contralesional vestibular nuclei (note that in simpler models such as the lamprey, UVD mainly generates imbalance in the RS system characterized by a net reduction of excitation in contralesional RS nuclei – Deliagina, 1997). Studies in UVD models demonstrated that acute disequilibrium results from a net reduction of spontaneous activity in ipsilesional CVN neurons due to the conjunction of reduced excitation because of sensory afferent loss and increased commissural inhibition from contralateral secondary vestibular neurons (Figure 4A).

Ipsilesional vestibular afferents were found to degenerate after the combined suppression of vestibular endorgans and Scarpa ganglion in anurans (Kunkel and Dieringer, 1994; Lambert et al., 2013) as well as after neurectomy in cats (Schuknecht, 1982) where the number of synaptic contacts onto ipsilesional CVN neurons drops by about one third (Raymond et al., 1991). Rapid and global metabolism reduction in ipsilesional CVNs was observed as soon as 3.5 h post-UVD in rats (Luyten et al., 1986; Paterson et al., 2006) and 2 h in cats (Maeda, 1988), indicating that spontaneous neuronal activity dramatically dropped in ipsilesional CVNs consecutive to afferent loss. In guinea pigs the proportion of ipsilesional ‘silent units’ after acute labyrinthectomy was estimated to be around 70% in the superior, lateral and medial nuclei (respectively SVN, LVN, and MVN), while the remaining active neurons displayed firing rates lower than controls (Ris et al., 1995; Ris and Godaux, 1998; Vibert et al., 1999a). Furthermore, neurons recorded from all ipsilesional CVNs still exhibited low firing responses to head rotation 1 week after the lesion (Ris et al., 1995). Patterns of silencing in ipsilesional CVNs were similarly reported from cats (Precht et al., 1966) and gerbils (Newlands and Perachio, 1990). In contrast, rostral MVN neurons on the lesioned side displayed an increased resting firing rate 4 h after UVD in rats (Yamanaka et al., 2000), which may explain the faster recovery from UVD observed in this species.

The cellular mechanisms responsible for ipsilesional CVN neuron depression early after UVD are not fully understood. A recent study in cats showed that the expression of small conductance potassium (SK) channels involved in post-discharge after-hyperpolarization rapidly rose in MVN, SVN, and LVN neurons during the first week following the lesion (Tighilet et al., 2019). SK channels expression was higher in contralesional CVNs and, although a neuronal phenotypic identification was lacking, both inhibitory and excitatory neurons were probably concerned. Such a disequilibrium in the expression of SK channels was proposed to affect notably commissural neurons and to account for balance loss between ipsi- and contralesional activity levels. Interestingly, it has been previously shown in cats (Cass and Goshgarian, 1990; Tighilet et al., 2007), guinea pigs (Rickmann et al., 1995) and rats (De Waele et al., 1996) that UVD induced both gliogenesis and increased astrocytic activity in CVNs in the first post-lesion days; given that astrocytes are essential components for extracellular glutamate and potassium homeostasis (reviewed in Walz, 2000), such a concomitant upregulation of SK channels in CVN neurons and increased astrocytic activity would ensure the CVN protection against hyperactivity-induced excitotoxicity and likely participate in the later bilateral activity re-equilibration (see below). In addition, as early as 3 days post-UVD, neurogenesis of neurons, mostly GABAergic, was also reported in cats to occur principally in the deafferented LVN, and to a lesser extent MVN and SVN (Tighilet et al., 2007; Tighilet and Chabbert, 2019), suggesting a possible role in the early decline of ipsilesional CVN activity (for a review on GABA effects on CVNs activity, see Gliddon et al., 2005). In addition, the strong reduction of ipsilesional spontaneous activity may consequently relieve contralesional CVNs from the commissural inhibitory control normally exerted by ipsilesional CVNs, making contralesional CVN neurons more spontaneously active. In turn, contralateral CVNs hence activated would exert in a stronger fashion their inhibitory (commissural) control on their ipsilesional counterparts, sustaining so the low excitability of ipsilesional CVN neurons. Though, a very recent study using optogenetics in mice suggested that an imbalance between the left and right MVN glutamatergic neurons might be the main source of deleterious effects of UVD on posture and locomotion, whereas GABAergic neuron selective activation had no clear impact (Montardy et al., 2021). Counter-intuitively with a reduction of activity in ipsilesional CVNs, mRNAs for NMDA receptor subunits NR1 and NR2C increased in the ipsilesional MVN of guinea pigs soon after UVD (Sans et al., 1997), which could suggest the instauration of a new complex balance between excitation and inhibition in CVNs following UVD; yet, because the nature of the cells expressing higher levels of NMDA subunit mRNAs was not identified, one may expect that such NMDA receptor overexpression could occur specifically in local inhibitory INs, increasing so their responsiveness to synaptic inputs and subsequently the inhibition of their target cells (as suggested by the work of Kitahara et al., 1995).

#### Associated Brain Structures

A UVD also affects the other CNS integrative structures implicated in the vestibular control of posture and locomotion. The loss of vestibular sensory information rapidly triggers widespread CNS adaptions, putatively in order to increase the weight of other sensory signals in the global sensory information used to operate posturo-locomotor functions. Supporting this idea, a very recent study in rats demonstrated that cortical somatosensory maps reorganized over the first hours following UVD, with bilateral expansion of the cutaneous receptive fields corresponding to the two hind paws and increased neuronal sensitivity to cutaneous stimulation (Facchini et al., 2021). Another example in rats comes from the cerebellum where a unilateral damage of the semicircular canal hair cells induced metabolic imbalance in the nodular cortex, characterized by an increase in glucose consumption on the ipsilesional side and a decrease on the contralateral side (Patrickson et al., 1985). This indicated that such a UVD had triggered activity imbalance between left and right nodules. Similarly, acute increases in nitric oxide (NO) synthesis enzymes were observed consecutive to UVD in the flocculus of rats (Kitahara et al., 1997a) and frogs *Rana esculenta* (Pisu et al., 2002), which indicated that plasticity occurred in this cerebellar subdivision particularly implicated in the integration of vestibular inputs (Figure 4A). While such increases were found bilaterally in these two species, they were always higher on the ipsilesional side. Furthermore, higher NO activity in cerebellar flocculus had been linked to concomitant reduction of the activation of contralesional CVNs and enhanced activity in the ipsilesional ones (Kitahara et al., 1999), which could participate in the later bilateral re-equilibration between CVNs (see below).

#### Spinal Cord

Surprisingly, given their fundamental role in locomotor and postural activity organization, very few is known about how spinal networks are acutely affected by a UVD. A functional hypo-activation of the ipsilateral soleus H-reflex has nevertheless been reported to occur shortly (less than 2 days) after a UVD in the alert baboon while contralateral reflexes were enhanced (Lacour et al., 1976). The ipsilesional reflex reduction was proposed to result mainly from hypo-excitability of both α and γ motoneurons, suggesting that spinal neurons are indeed affected in an early time window following UVD.

### Late Unilateral Vestibular Deprivation-Induced Modifications – The ‘Vestibular Compensation’

Early events that evoke vestibular imbalance are followed, with variable timelines depending on the considered species, by further plasticity mechanisms leading to a nearly full recovery of static head and trunk posture while dynamic deficits are never fully restored. Supposedly, these mechanisms constitute the substrate for the so-called ‘vestibular compensation,’ taking place in every different structure mentioned above and resulting in the re-equilibration of CVN neuronal resting discharge on the two sides (Ris et al., 1997; Vibert et al., 1999b). At the CVN level, the restoration of balance results principally from modifications in the connectivity and efficacy of both sensory and commissural vestibular connections associated with changes in intrinsic properties of secondary vestibular neurons on the side of the lesion (Figure 4B). Such a combination of pathway reorganization and post-lesional cellular plasticity was suggested to account for the correction of static syndromes (see Lambert and Straka, 2012).

#### Sensory Substitution

Restoration of functional balance would be enhanced notably by substituting the lost vestibular signals with visual (e.g., Carcaud et al., 2017) and proprioceptive information (e.g., Jamali et al., 2014), which are also integrated by brainstem nuclei.

Interaction between visual and vestibular afferent signals were particularly well studied in the context of vestibulo-ocular reflexes where visual stimuli were found to regulate the reflex gain acting directly on secondary vestibular neuron intrinsic and synaptic properties (Carcaud et al., 2017). Whereas cellular mechanisms were much less analyzed in the context of posture control, several studies nevertheless demonstrated the substantial participation of visual stimuli in UVD compensation. In the lamprey, for example, UVD-evoked RS imbalance can be counteracted by stimulating the contralesional eye. Indeed, because ipsilesional RS nuclei still receive vestibular signals arising from the intact contralateral endorgans, the visually generated synaptic inputs impacting only upon contralesional RS nuclei restores the bilateral RS excitation balance (Deliagina, 1997). In contrast, suppressing movement-related visual information in UVD cats, by housing them for 2 weeks in a stroboscopic environment (Zennou-Azogui et al., 1996) or in darkness (Putkonen et al., 1977), was found to delay vestibular compensation. Similarly, stabilizing the visual environment with respect to the animal’s head delayed as well the occurrence of compensation in UVD baboons (Lacour and Xerri, 1980), suggesting a role for the motion-related visual flux in correcting activity balance between bilateral CVNs. Vision seems to be fundamental as well in maintaining compensation in cats since UVD cats put into darkness after vestibular compensation display the characteristic head tilt of UVD animals, an effect that rapidly disappears after return into light (Putkonen et al., 1977). However, a recent meta-analysis of vestibular compensation bibliography in UVD animals revealed that, overall in mammals, vision has a limited impact on the increase in intrinsic excitability of ipsilesional CVN neurons (Wijesinghe and Camp, 2020) and the homeostatic recovery of bilateral CVN symmetry.

In contrast, lifting guinea-pigs from the ground early after UVD dramatically delayed the recovery of postural symmetry (Schaefer and Meyer, 1973). Similarly, restraining baboons made UVD-induced postural disorders to be compensated much later (Lacour et al., 1976). These results indicate that locomotor activity facilitates vestibular compensation processes, likely by generating movement-related proprioceptive and cutaneous information used to feed deafferented CVNs. Moreover, the increase in density (Dieringer et al., 1984) and weight of proprioceptive projections ascending from the spinal cord onto vestibular nuclei (Sadeghi et al., 2011) also seems to be a key element in maintaining posture correction in compensated UVD animals. Indeed, deprivation of brainstem nuclei from sensory feedback by suppressing ground contact or disconnecting ascending lateral tracts including spino-vestibular fascicle triggered static symptoms reappearance after compensation in rats and guinea pigs (Azzena, 1969; Jensen, 1979b; Schaefer et al., 1979).

Although reported from distinct animal species these results taken together suggest that an interplay between vision and proprioception may be the key mechanism involved in substitution for the lacking vestibular sensory information in UVD animals. It is noteworthy, however, that the time course of vestibular sensory deprivation directly impacts the ability to use visual cues for motor deficit compensation, as observed in human patients where a fast unilateral loss of vestibular information results in lesser visual dependence and stronger inability to maintain postural stability (Tjernström et al., 2019). In addition, after partial vestibular lesion the modification of both the organization and weight of residual vestibular afferents onto CVN neurons (Goto et al., 2002) as well as the possibility of peripheral plasticity in vestibular endorgans (Gaboyard-Niay et al., 2016) certainly participate in CVN adaptation and subsequent posturo-locomotor recovery (Cassel et al., 2018). Indeed, the degree of proprioceptive information utilization to control posture in UVD patients increase with their inability to use residual vestibular information (Peterka et al., 2011). To date, however, the cellular and synaptic mechanisms leading CVN neurons to adapt the relative weight of various sensory input signals in order to restore body symmetry remain unraveled.

#### Central Vestibular Neurons

A variety of post-lesional plasticity cellular processes were described in various vertebrate species that were proposed to account for the restoration of resting activity in ipsilesional vestibular nuclei and the resultant vestibular system re-balancing. Indeed, long-term vestibular compensation results in the re-equilibration of resting activity between CVNs on the two sides (e.g., Darlington et al., 1989; Newlands and Perachio, 1990; Beraneck et al., 2003; Beraneck and Idoux, 2012) or between bilateral RS nuclei in the lamprey (Pavlova et al., 2004). In guinea pigs developing post-UVD compensation, intracellularly recorded ipsilesional MVN neurons exhibited pacemaker properties conferring the inherent ability to generate spontaneous activity (Darlington et al., 1989). This observation indicated that compensation did not result exclusively from modifications of input synapses, notably sensory input synapses (see above), but also from plastic changes in CVN neurons’ intrinsic properties (Vibert et al., 1999b; Beraneck and Idoux, 2012). In cats, the overexpression of SK channels rapidly observed after UVD faded with time during the compensation process and re-equilibrated between CVNs on both sides (Tighilet et al., 2019). Because intrinsic properties could be modulated by the variety of neurotransmitters and neuromodulators environing CVNs (Darlington et al., 2002; Beraneck and Idoux, 2012), the input synapses that reorganize during vestibular compensation, including sensory ones, might enhance pacemaker properties and concomitantly restrict SK expression in order to ‘restart’ CVN activity on the lesioned side. Investigations in brainstem slices from guinea pigs at 1 month post-UVD demonstrated that both the spontaneous resting discharge and the discharge evoked by intracellular current injection were increased in the two types of secondary vestibular neurons in ipsilesional CVNs (with a more marked effect in type-B compared to type-A neurons) and that type-B neurons exhibited membrane properties closer to type-A neuron properties (Beraneck et al., 2003).

Cellular and synaptic plasticity initially coincides with several waves of protein kinase-dependent induction of immediate early genes (IEGs), genes that are activated rapidly and transiently in response to a variety of cellular events (for reviews: Beckmann and Wilce, 1997; Minatohara et al., 2016). IEGs induction (e.g., transcription factors *fos* and *zif-268*; Lacour and Tighilet, 2010) was identified in CVNs of UVD cats to occur between 3 h and 7 days after the lesion. Of importance, injecting protein kinase inhibitors in the fourth ventricle of rats dramatically delayed the recovery from UVD-induced motor symptoms (Balaban et al., 1999), suggesting that activating IEGs is necessary to trigger subsequent vestibular compensation processes. Cellular and synaptic plasticity mechanisms are also mirrored by metabolic activity recovery in deafferented CVNs (see Dieringer, 1995), associated with an upregulation of mitochondrial proteins in rats (Paterson et al., 2006). These observations suggest that compensation relies on processes actively engaging several intracellular cascades.

#### Network Adaptation – Role of Inhibitions

Besides changes in intrinsic neuronal properties, network reorganization was also reported to occur during the compensation process that led to the functional restoration of symmetrical motor activities. As mentioned above, both reactive neurogenesis and gliogenesis were reported to take place soon after UVD. Interestingly, an antimitotic treatment applied during the first 3 weeks post-UVD impairs both neuron and microglia proliferation and drastically delays behavioral recovery (Dutheil et al., 2009), suggesting that generating new cells is a prerequisite for behavioral compensation to occur. In addition, upregulation of proteins involved in axon growth and guidance and in mitochondrial metabolism 1 week after the lesion was reported in UVD rats (Paterson et al., 2006), whereas the early process of activity restoration has been found not to depend on protein synthesis in guinea pigs (Ris et al., 1998). Again, such differences illustrate the variety of mechanisms observed in distinct species. Whereas the role of gliogenesis has not been investigated in this process, neurogenesis, especially GABAergic neuron genesis, could participate in the restoration of bilateral balance between deafferented and intact CVNs (see Tighilet and Chabbert, 2019).

In the adult cat, about 60% of the new neurons in ipsilesional CVNs survive and display markers of GABAergic INs, which could represent new local and/or commissural INs projecting onto contralateral CVNs (Tighilet et al., 2007). During early stages of vestibular compensation in rats, the responses of secondary vestibular neurons to exogenous GABA application was found to be downregulated in deafferented CVN neurons while they were enhanced on the contralesional side (Yamanaka et al., 2000), and GABA agonists were shown to reduce UVD-induced motor symptoms (Magnusson et al., 2000). In parallel, modifications of expression levels of both GABA receptors and K^+^/Cl^–^ co-transporter KCC2 were also reported in CVN neurons; however, GABA receptors were bilaterally upregulated, whereas KCC2 was decreased on the ipsilesional side and increased on the contralesional one (Dutheil et al., 2016). KCC2 regulates the chloride transmembrane gradient in neurons and exhibits different levels of expression during development (Schulte et al., 2018). The low expression of KCC2 in early developmental stages does not counteract chloride accumulation in neurons (caused by the other ion cotransporter NKCC1), and GABA receptor activation results in the neuron to be depolarized because of chloride anions exiting the cell. In contrast, KCC2 upregulation during later development strongly decreases the intracellular concentration of chloride ions and so, maintains the chloride gradient in such a way that GABA receptor activation now induces anion entry and neuronal hyperpolarization. Thus, in a UVD context, KCC2 overexpression on the intact side would make contralesional CVN neurons ‘more sensitive’ to inhibition while KCC2 downregulation in ipsilesional CVN neurons would cause locally the GABAergic (as well as the glycinergic – Vibert et al., 2000) neurotransmission to be less inhibitory, or even excitatory (Dutheil et al., 2016). As a consequence, switching GABAergic influence from being inhibitory to being excitatory could represent a homeostatic way to maintain a sufficient excitation level in ipsilesional CVN neurons and further contribute to deafferented neuron survival in the absence of their principal excitatory inputs. Moreover, maintaining excitability of ipsilesional CVN neurons by reducing synaptic inhibition and increasing synaptic excitation may also trigger the changes that are observed in CVN neuron membrane properties, since synaptic inhibition drops before intrinsic properties are modified (Vibert et al., 2000). Interestingly, UVD cats treated with intraventricular injection of brain-derived neurotrophic factor (BDNF) early after the vestibular lesion exhibited increased GABAergic neuron neurogenesis on the ipsilesional side and no KCC2 modification on either side (Dutheil et al., 2016), suggesting that increasing the number of GABAergic neurons would be sufficient to trigger behavioral restoration. This further suggests that KCC2 specific alteration on the two sides may constitute a rapidly acquired but transient state of CVN neurons to restore balance acutely, before inhibition is re-equilibrated in ipsilesional CVNs by addition of new functional inhibitory neurons.

Such modifications are thought to impact all inhibitory inputs that converge onto CVN neurons, i.e., local inhibitory INs, commissural influences and inhibitory projections from the cerebellum (Dieringer and Precht, 1979b; Yamanaka et al., 2000). Long ago, commissural connections were proposed to be primordial in maintaining CVN bilateral balance, and commissural integrity was demonstrated to be necessary for the restoration of such balance in UVD cats (Precht et al., 1966; Maeda, 1988). In guinea pigs as well, bilateral balance of CVNs is ensured by the bilateral equilibrium of commissural inter-connections (Ris and Godaux, 1998), and restoration of this equilibrium after a UVD is necessary for later behavioral recovery (Olabi et al., 2009). Another argument in favor of a fundamental implication of commissural cross-inhibitions in vestibular compensation was the demonstration in cats that facilitating histamine release enhanced recovery of the affected motor functions (Tighilet et al., 2006). Indeed, histamine released from tuberomammillary cells (in the posterior hypothalamus) onto CVN neurons affects commissural inhibitory pathways and dramatically reduces the response of secondary vestibular neurons to sensory-evoked GABA release (Olabi et al., 2009). It had been shown in UVD cats that the histamine synthesis enzyme was upregulated in the ipsilesional tuberomammillary nucleus during the first week following the vestibular lesion and then slowly returned to control values in a time window consistent with the course of electrophysiological (CVN re-balancing) and behavioral (motor symptoms) recovery (Tighilet et al., 2006). In contrast, it was proposed in terrestrial frogs that vestibular compensation rather involved an increased participation of the excitatory commissural connections from intact to deafferented CVNs (Dieringer and Precht, 1979a; Kunkel and Dieringer, 1994), while inhibitory regulation came principally from the cerebellum (Dieringer and Precht, 1979b).

#### Cerebellar Changes

Unilateral vestibular deprivation-induced plasticity in cerebellar nuclei is also likely involved in compensation processes in mammals, although the exact degree of cerebellum implication remains under debate (Smith, 2020). In UVD rats, a NO increase in the cerebellar flocculus had been correlated with *fos* immunolabeling intensification in ipsilesional and decline in contralesional CVNs, respectively (Kitahara et al., 1997a,^1999^), illustrating the relative overactivity in deafferented CVNs compared to intact ones when cerebellar NO activity rose. Interestingly, blocking NO synthase in the flocculus early after the vestibular lesion resulted in the opposite ratio (Kitahara et al., 1999), due to the UVD-induced asymmetrical activation of CVNs (see above), and caused retardation in motor recovery (Kitahara et al., 1997a). Furthermore, destroying the ipsilesional flocculus prevented any later increase in membrane excitability of deafferented MVN neurons (Kitahara et al., 1997b; Johnston et al., 2002). These results suggest that plasticity in the GABAergic transmission from Purkinje cells plays a significant role in the bilateral re-balancing of CVNs (Figure 4B), likely during the initial phase of vestibular compensation since floccular NO expression has been found to fade afterward during the compensation process (Kitahara et al., 1997a). How such plasticity is triggered in vestibulo-cerebellar networks in response to vestibular asymmetry remains largely unexplored. Nevertheless, the higher expression of acetylcholine described in UVD cat secondary vestibular neurons (Tighilet and Lacour, 1998), which could increasingly activate cholinergic receptors on both cerebellum granule and unipolar brush cells (Jaarsma et al., 1997), might constitute one of the initial cellular events triggering plasticity in cerebellar neurons. Plasticity might result as well from the inactivation of kinases constitutively expressed in Purkinje cells (Barmack et al., 2001) and the return to normal expression levels of glutamate receptor subunit δ2 after its initial UVD-induced drop (Kitahara et al., 1998). Such a substantial implication of the cerebellum, and that of other higher brain structures such as the hippocampus or inferior olive (Llinás et al., 1975; Wu et al., 2010; for a review, see Darlington and Smith, 2000), suggests that compensation of the permanent loss of vestibular sensory information on one side finally consists of the combined bilateral re-equilibration of CVN activity and development of new motor strategies (Curthoys and Halmagyi, 1999).

#### Spinal Cord

Again, almost no dedicated investigations were performed about spinal network alterations and their putative role during post-UVD motor recovery processes, and the very few data available come from indirect observations. After vestibular compensation in UVD guinea pigs, for instance, targeted disruption of the ascending spinovestibular fascicule induced the loss of bilateral CVN balance, demonstrating that the integrity of spinal ascending information was necessary to maintain the compensated CVN function (Jensen, 1979b). In addition, in already compensated animals but not in intact controls the same spinal lesion also evoked subsequent postural asymmetries that persisted several days after the surgery (Jensen, 1979a), demonstrating that substantial spinal plasticity occurred that was revealed by the disconnection from brain centers. A recent longitudinal study of gait recovery in UVD humans reported a progressive recovery of step length and ipsilateral stance duration, which was proposed to be the behavioral expression of new motor strategies to compensate for a unilateral vestibular loss (Chae et al., 2021). However, both could also simply reflect some spinal network adaptation, functionally reorganizing in a motor activity-dependent process both the local sensory-motor loops and propriospinal connections to ensure maximum vertical stability of the body. All these observations suggest that during the vestibular compensation process, plasticity certainly also occurs within spinal sensory-motor networks. Indeed, behaviorally relevant sensory-motor plasticity was reported in the spinal cord of chronic UVD terrestrial frogs (Straka and Dieringer, 1995) where neck control recovery initiated before CVN re-balancing and depended on the bilateral enhancement of local cervical reflexes, with stronger effect on the ipsilesional side.

### Unilateral Vestibular Deprivation-Induced Developmental Adaptation of Motor Networks

**FIGURE 5:**
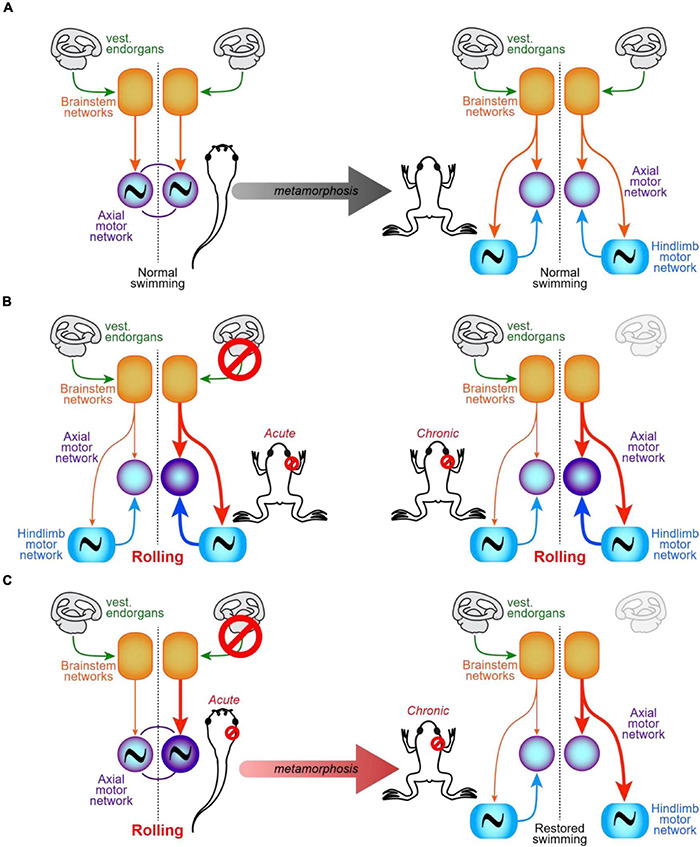
Functional organization of the spinal posturo-locomotor network in the intact and vestibulo-lesioned *Xenopus*. **(A)** Schemes illustrating the symmetrical organization of the most basic brainstem/spinal cord sensory-motor network responsible for the control of locomotion and posture in intact pre-metamorphosis tadpole (left) and post-metamorphosis juvenile (right) *Xenopus* (adapted from Beyeler et al., 2008). **(B)** Acute (left) and chronic (time interval equivalent to metamorphosis duration; right) effects of a right-side UVD in the juvenile *Xenopus*. Note that vestibular endorgans/Scarpa ganglion removal generates a net ipsilesional over-excitation of the spinal network, which results in a permanently impaired locomotor behavior (i.e., rolling swimming toward the lesion side; adapted from Beyeler et al., 2013). **(C)** Acute UVD (left) triggers spinal imbalance and rolling behavior in tadpoles. In contrast, after the animals had metamorphosed (right) normal swimming is restored although bilateral descending commands remain imbalanced. Recovery is allowed by a symmetrical activation of the postural axial network resulting from the construction, during metamorphosis, of an asymmetrical propriospinal posturo-locomotor network (adapted from Beyeler et al., 2013). Thin lines, normal activation; thick lines, hyper-activation; vest., vestibular.

The specific interruption of semi-circular canal signals on one side early after birth delayed but did not prevent acquisition of motor abilities in rats (Geisler et al., 1997). However, no clear modifications of the temporal organization of locomotion were observed while trunk stability remained non-optimal for a longer time, thus suggesting that retardation might result from a deficit in dynamic coordination between locomotion and posture. Whether such coordination deficits resulted exclusively from the persistent default in descending commands or also from a lack of spinal network adaptation was not investigated. In fact, only few studies have analyzed the impact of a UVD on the subsequent development of spinal neural networks involved in the control of posture and locomotion, and the clearer results come from studies in the aquatic anuran *Xenopus laevis* (Figure 5).

In the larval *Xenopus*, as in all vertebrates, a UVD causes a loss of balance between ipsi- and contralesional CVNs; however, contrary to the other animal models, this imbalance is never compensated for and even subsists during metamorphosis to be similarly present in the post-metamorphic frog (Lambert et al., 2008, 2013; Beyeler et al., 2013). Interestingly, UVD effects on posture are identical whether it is performed before (tadpole stage) or after metamorphosis (juvenile stage), with a typical head twist and trunk curvature toward the lesioned side that remains uncorrected, likely because of the lack of ground-related sensory feedback in this purely aquatic animal. Performed at either developmental stage, UVD also similarly evoked locomotor deficits, in the form of a constant rolling behavior toward the lesion side (Beyeler et al., 2013). However, whereas swimming remained impaired in animals lesioned after metamorphosis, those lesioned before metamorphosis recovered near-normal locomotion after metamorphosis completion (Beyeler et al., 2013) whilst brainstem descending commands stayed permanently unbalanced (Lambert et al., 2013). Both *in vivo* and *in vitro* analyses of the spinal network responsible for dynamically coordinating posture and propulsion during swimming in post-metamorphic frogs demonstrated dramatic alteration in juveniles that have been lesioned before metamorphosis, compared to control animals or juveniles in which UVD had been performed after metamorphosis (Beyeler et al., 2013). Whereas both control (Beyeler et al., 2008) and juveniles vestibulo-lesioned after metamorphosis possessed functionally symmetrical spinal posturo-locomotor networks (Figures 5A,B), juveniles that were lesioned before metamorphosis exhibited an asymmetrical network, characterized by the functional loss on the lesioned side of the lumbo-thoracic coordination subduing axial postural muscles to the appendicular propulsive system (Figure 5C). Biomechanical models demonstrated that this simple alteration was sufficient to counteract the unbalanced brainstem descending commands evoked by the UVD. Indeed, UVD-induced disequilibrium consisted of a relative increase of the commands descending into the ipsilesional side of the cord, notably because of the loss of ipsilesional tangential neurons that normally project into the contralesional hemicord (Lambert et al., 2013). Hence, the ‘developmental suppression’ of ipsilesional lumbo-thoracic coordination results, during swimming, in the loss of propriospinal excitation onto postural MNs specifically on the lesion side, while contralateral MNs still receive this ascending excitation. As a result, during swimming the increased descending command on the ipsilesional side is compensated for by the absence of ascending propriospinal excitation, while the reduced descending command on the contralesional side is compensated for by the persistence of (and thus, relative to the other hemicord, increase in) ascending propriospinal excitation originating from the lumbar CPG. Altogether, these two opposite asymmetries lead to re-balancing activity in the spinal postural system during locomotion and allow normal (non-rolling) swimming in juveniles that underwent a UVD before metamorphosis.

Whether such spinal reorganizations are specific to *Xenopus* metamorphosis or occur similarly in other vertebrate species remains to be determined. Although very few investigations have been made in this direction, several indirect results tend to indicate that UVD-triggered spinal network adaptations may constitute a general feature in a majority of vertebrates. Hence, adult UVD rats that display severe postural defects at rest and dramatically unstable locomotion at low speed of walking, exhibit a more stable body balance with increased locomotor velocity (Rastoldo et al., 2020). Similarly, a running UVD dog shows less deviation from the normal path and less imbalance than during walking (Brandt et al., 1999). Interestingly, the exact same observations were made in patients with acute vestibulopathy in which walking strides were more regular and body balance more accurate at high velocity than at low speed (Brandt et al., 1999; Schniepp et al., 2012; reviewed in Akay and Murray, 2021). Two explanations could account for such a ‘speed-dependence’ of posture and gait in UVD subjects. First, this could result from decreasing the weight of vestibulospinal control onto spinal networks during high velocity locomotion as proposed by Akay and Murray (2021). Indeed, a study in healthy human subjects showed that gait deviation induced by a vestibular galvanic stimulation was reduced by half during running compared to walking, suggesting that higher locomotor speed exerted stronger inhibitory control on descending vestibulospinal commands (Jahn et al., 2000). In this study, however, galvanic stimulation mimicked sudden shifts of head position while locomotion was already ongoing, generating disrupting vestibular signals thus superimposed on still symmetrical tonic descending influences from gravity detectors. Of course, it is totally different in subjects with acute vestibular imbalance, where the basal tonic influence is no more symmetrical. An alternative explanation would arise from the possibility that, as demonstrated in juvenile *Xenopus* (Beyeler et al., 2008), the spinal posturo-locomotor network is intrinsically able to organize postural controls that are dynamically adapted to the ongoing propulsive motor program. In this context, locomotion and dynamic control of posture would be principally based on highly automated programs organized by spinal networks coordinated through propriospinal interconnections and under control of both tonic and dynamic supraspinal modulation (e.g., Olechowski-Bessaguet et al., 2020). The relative weight of propriospinal coordination vs. descending commands would then determine how the dynamic control of posture would be achieved (Beyeler et al., 2013): stronger is the spinally generated motor command, lower is the relative weight of dynamic descending inputs. Whether such balance is purely orchestrated at the spinal synaptic level or rather/additionally involves direct modulation of sensory inputs at the supraspinal level (Le Ray et al., 2010; Chagnaud et al., 2015; Straka et al., 2018) still has to be determined. Thus, running may rely much less on dynamic descending inputs than slow walking does. Yet, after UVD this would require precise alterations of the spinal posturo-locomotor network taking into account the persistent disequilibrium in tonic descending influences. However, contrary to what was described in the metamorphosing *Xenopus* where propriospinal adaptation result from developmental processes, it is likely that such alterations in mammals would rather involve plastic adaptations closer to those consecutive to a unilateral SCI. The existence of such UVD-triggered spinal reorganization, whether it is developmental or adaptive, confronts the classical conclusions about UVD effects on the control of posture (e.g., Vidal et al., 1993) which were drawn from studies restricted to the control of the so-called ‘static’ posture.

## Conclusion

In pluricellular organisms, the development of symmetry relies on both animal genetics and inter-cell communication (Allard and Tabin, 2009; Moubayidin and Østergaard, 2015), and symmetries appear to be the most appropriate patterns in the evolution of animal body arrangement (Holló, 2015). Spatial coordination between limb muscles is organized symmetrically from pelvis to ankle, which ensures best and less energy consuming locomotion (Saunders et al., 1953), and patients with developmental coordination disorders present asymmetrical gait and impaired locomotor efficacy compared to neurotypical subjects (Wilmut et al., 2017). Thus, due to the bilaterally symmetrical arrangement of their biomechanical apparatus, animals require a similar symmetry in the structure of their basic sensory-motor circuits, from development to adulthood, to achieve accurate locomotor and postural controls (Farel and McIlwain, 2000; Holló, 2015).

Here, we have reviewed several (non-exhaustive) examples of behavioral consequences of accidental breaks in fundamental symmetry of the sensory-motor CNS throughout the vertebrate kingdom, as well as plasticity that is elicited to face the motor deficits generated by such symmetry failures. We have seen that sensory-motor networks can cope quite remarkably well with the information remaining in injured and incapacitated motor or sensory nervous structures. As a result, plasticity mechanisms distributed throughout the CNS orchestrate the reestablishment of bilateral neural equilibrium, i.e., the best possible functional symmetry within spared neuronal networks and pathways, whether injury was of central or sensory origin. Such a generalization of homeostatic plasticity leads to the conclusion that symmetry in the motor CNS is a functional necessity and not merely an ‘experimentally impossible to verify but plausible just-so’ of evolution (see Cooke, 2004). Indeed, all reactions at cellular, synaptic or network level initiated in response to unilateral neural injuries tend to restore the lost functional symmetry in the sensory-motor CNS. In addition, adaptations are not restrained but involve the sensory-motor CNS as a whole, and behavioral recovery depends on the interplay between all neural actors. Nevertheless, every single elements comparably possesses the ability to adapt its operation in response to an accidental loss of neural symmetry, as observed for instance in cats where, after complete transection, spinal circuitry retains the ability to adjust to ulterior cutaneous denervation (Bouyer and Rossignol, 2003).

Finally, what does determine symmetry in the sensory-motor CNS, during development and afterward? It is commonly admitted that body symmetry, as well as natural symmetry breaks, are strictly encoded, the genome being capable of selecting the adequate scheme according to the animal’s behavioral requirements (Holló, 2015). Yet, it is not clear what kind of symmetry is encoded when we consider posturo-locomotor functions. According to the general rule enacted in biology (Holló, 2015; Moubayidin and Østergaard, 2015), one may answer that genes encode the structural symmetry of the sensory-motor CNS. However, based on studies reviewed above illustrating the sensory-motor CNS extraordinary capacity to adapt and change its organization to restore the most accurate function after an accidental loss of central or sensory symmetry, we propose that function (here, posturo-locomotor control) rather than CNS strict anatomical arrangement may be the fundamental target of networks developmental assembly. Especially, experiments performed in metamorphosing *Xenopus* (Beyeler et al., 2013) strongly support this view since altering symmetry in vestibular sensory inputs during posturo-locomotor network metamorphic construction triggered the establishment of an asymmetrical spinal motor network able to ensure an appropriate function in a still sensory-deprived, post-metamorphic animal.

## Author Contributions

DLR and MG wrote the manuscript. Both authors contributed to the article and approved the submitted version.

## Conflict of Interest

The authors declare that the research was conducted in the absence of any commercial or financial relationships that could be construed as a potential conflict of interest.

## Publisher’s Note

All claims expressed in this article are solely those of the authors and do not necessarily represent those of their affiliated organizations, or those of the publisher, the editors and the reviewers. Any product that may be evaluated in this article, or claim that may be made by its manufacturer, is not guaranteed or endorsed by the publisher.
